# Echocardiographic alterations in a child with cow’s milk allergy: a case report

**DOI:** 10.1186/1752-1947-6-299

**Published:** 2012-09-13

**Authors:** Giuseppe Di Cara, Maria Giulia Berioli, Anna Biscarini, Claudia Soldani, Piera Abate, Eleonora Ugolini, Giusiana Allocca, Maddalena Milioni

**Affiliations:** 1 Servizio di Immuno-Allergologia Pediatrica, University of Perugia, Perugia, 06100, Italy; 2 Division of Pediatrics, Azienda Ospedaliera di Perugia, Perugia, 06100, Italy

**Keywords:** Cow’s milk allergy, Kawasaki disease

## Abstract

**Introduction:**

Cow’s milk allergy is the most frequent food allergy in Europe and western countries and shows a wide spectrum of clinical features, including atopic dermatitis and gastrointestinal disease. To the best of our knowledge, this report is the first to describe Kawasaki disease-like clinical features and echocardiographic alterations which resolved after a cow’s milk-free diet.

**Case presentation:**

We report a case of a 9-month-old Caucasian girl with atopic dermatitis who developed clinical features commonly present in Kawasaki disease (erythematous skin rash, non-exudative conjunctivitis, fissured lips and neck lymph nodes), together with mild echocardiographic alterations (perivascular brightness, pericardial effusion) in the absence of fever. These features resolved within 2 weeks after the beginning of a cow’s milk-free diet.

**Conclusion:**

Kawasaki disease has recently been considered a possible risk factor for subsequent allergic disease secondary to immune dysfunction. This case report suggests that the immune-related alterations which are commonly present in allergic patients could be similar to the antigen-related immune response in Kawasaki disease and thus could lead to similar clinical features.

## Introduction

Cow’s milk allergy (CMA) is an immunologically-mediated adverse reaction to several allergenic proteins present in cow’s milk which affects approximately 5% to 7% of children in the first years of life and 2% to 3% of adults [1]. Immunological mechanisms leading to this food allergy, which is the most frequent food allergy in Europe and western countries, include immunoglobulin E (IgE)-mediated hypersensitivity and cellular immune response. CMA is commonly a disease of early infancy, with children developing symptoms a few weeks after the introduction of cow’s milk-based formula into the diet. The most common features of CMA in the first years of life include atopic dermatitis and gastrointestinal diseases such as food protein-induced enterocolitis and allergic eosinophilic gastrointestinal disorders [2]. We report on a patient with atopic dermatitis and previously undiagnosed cow’s milk allergy who was admitted to our hospital with clinical, laboratory and echocardiographic findings frequently present in Kawasaki disease (KD). The absence of fever, a leading feature in the diagnosis of KD, excluded such a diagnosis, and the presence of high specific serum IgE towards cow’s milk, together with the negativity of other tests for several infectious diseases, suggested the role of cow’s milk allergy in the development of these symptoms.

## Case presentation

A 9-month-old Caucasian girl was admitted to our hospital for the presence of a persistent skin rash and redness of the eyes. Her medical history was positive for mild atopic dermatitis present since 6 months of age. In the 3 weeks prior to admission, she did not have fever. Her physical examination showed an erythematous rash involving the trunk and limbs, fissured lips, erythema of oral mucosa, bilateral conjunctival injection without eye discharge and laterocervical lymph nodes. The respiratory and abdominal examinations were normal. The cardiac evaluation showed tachycardia with normal heart sounds. The laboratory results revealed a white blood cell count of 8500/mm^3^ (normal range: 3600 to 9600/mm^3^) with normal differential counts, hemoglobin level of 10.7g/dl (normal range: 11.5 to 14.5g/dl), platelet count of 376,000/mm^3^ (normal range: 150,000 to 350,000/mm^3^), erythrocyte sedimentation rate of 35mm/hour (normal range: 5 to 25mm/hour), C-reactive protein level 1.5mg/dl (normal range: 0 to 1mg/dl), albumin level 2.8g/dl (normal range: 3.5 to 5.5g/dl), alanine aminotransferase 24U/L (normal range: 15 to 45U/L), aspartate aminotransferase 27U/L (normal range: 20 to 55U/L), γ-glutamyl transferase 59U/L (normal range: 0 to 35U/L), direct bilirubin 0.4mg/dl (normal range: 0.3 to 1mg/dl), IgG 589mg/dl (normal range: 380 to 790mg/dl), IgA 34mg/dl (normal range: 20 to 120mg/dl). Serum-specific IgE against cow’s milk proteins were positive (12.5kU/L; normal values: 0 to 0.35kU/L).

**Figure 1:**
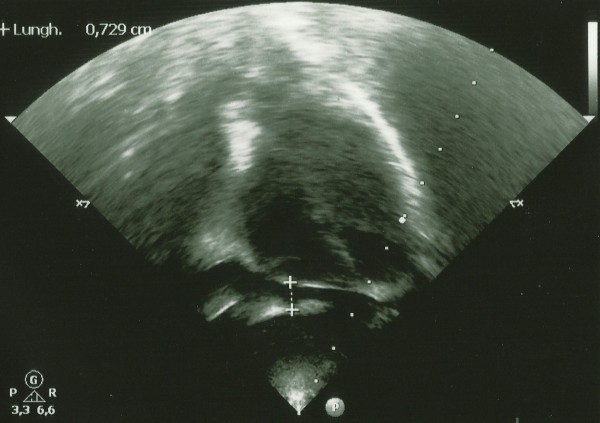
Echocardiographic findings: pericardial effusion and perivascular brightness of the proximal left descending coronary artery.

The blood culture, urine culture and serology for Epstein-Barr virus, cytomegalovirus, measles, rubella, parvovirus and adenovirus were all negative. Antistreptolysin antibodies were negative. Her chest radiogram was normal. Echocardiography performed at admission showed mild pericardial effusion and perivascular brightness of the proximal left descending coronary artery (Figure 1).

Because of the absence of fever before and during the admission, the diagnosis of KD was excluded. A cow’s milk-free diet was started 2 days after admission, which led to resolution of the clinical findings and normalization of the laboratory findings after 1 week. A second echocardiography, performed 2 weeks after the beginning of the diet, was normal. During the 2 weeks of the infant’s admission, she never presented fever, and no interventions other than the cow’s milk-free diet were necessary.

## Discussion

The relationship between KD and atopy has been evaluated in a large cohort study which evidenced a significantly higher incidence of allergic rhinitis and atopic dermatitis in children who have had KD [3]. Matsuoka *et al*. proposed that the genetic predisposition to atopy could be associated with a higher susceptibility to KD. In addition, patients with KD tend to have a higher risk of developing atopic dermatitis and allergic rhinitis. Recently, a study of 93 sibling pairs supported the hypothesis of an inherited genetic pattern which increases the risk both of KD and atopy, secondary to an immune disequilibrium which leads to an abnormal inflammatory response to different trigger events [4].

Our report of a child who presented several clinical features of KD, together with mild cardiac echocardiographic alterations, both of which resolved after a cow’s milk protein-free diet, underlines the relationship between atopy and KD.

Although most of the clinical features we evidenced were non-specific, as can be present in several infectious diseases (although in this child serological tests were negative for all pathogens causing similar findings), the association with echocardiographic alterations has not been reported previously in children with food allergies.

Our data support the hypothesis that the same heritable genetic factors that induce a specific pattern of altered or impaired immune maturation could increase the risk of developing both KD and allergy.

## Conclusions

KD has been considered a possible risk factor for subsequent allergic disease secondary to immune dysfunction. We suggest that immune-related alterations which are commonly present in allergic patients such as those with CMA may be similar to the antigen-related immune response in KD and thus could lead to similar clinical features. To the best of our knowledge, this report is the first to describe KD-like clinical features and echocardiographic alterations which resolved after a cow’s milk-free diet.

## Consent

Written informed consent was obtained from the patient’s parents for publication of this case report. A copy of the written consent is available for review by the Editor-in-Chief of this journal.

## Competing interests

The authors declare that they have no competing interests.

## Authors’ contributions

GDC, MGB, AB and CS evaluated the pat+ient during admission and evaluated her during the follow-up period. PA, GA, EU and MM were major contributors to the writing of the manuscript. All authors read and approved the final manuscript.
